# Angiomyolipome épithélioïde rénal mimant un carcinome rénal

**DOI:** 10.11604/pamj.2016.24.99.8557

**Published:** 2016-05-27

**Authors:** Hanen Bouaziz, Ramzi Khiari, Mohamed Dridi, Samir Ghozzi, Nawfel Ben Rais

**Affiliations:** 1 Service de Chirurgie Carcinologique, Institut Salah Azaiez, Tunis, Tunisie; 2 Sercice d'Urologie, Hôpital Militaire, Tunis, Tunisie

**Keywords:** Angiomyolipome, néphrectomie, tomodensitométrie, Angiomyolipoma, nephrectomy, computed tomography

## Abstract

L'angiomyolipome épithélioide est une forme rare d'angiomyolipome à potentiel malin, considéré récemment par l'OMS comme une entité à part dans la classification des tumeurs rénales. Cette lésion pose un problème dans le diagnostic différentiel avec les carcinomes à cellules claires. Il n'y a pas de critère spécifique clinique ou radiologique caractérisant cette tumeur. L'immunohistochimie en révélant la positivité des cellules épithélioide au marqueur HMB45 est essentielle au diagnostic. Le traitement doit être discuté en concertation pluridisciplinaire.

## Abstract (en)

Epithelioid angiomyolipoma is a rare form of potentially malignant angiomyolipoma, recently considered separate entity by the World Health Organization classification of renal tumors. This lesion poses a problem in differential diagnosis with clear cell carcinomas. There are no clinical or radiological specific criteria that characterize this tumor. Immunohistochemistry revealing epithelioid cells with positive HMB45 marker is essential for diagnosis. Treatment should be discussed during the multidisciplinary consultation.

## Introduction

Les angiomyolipomes (AML) sont des tumeurs bénignes mésenchymateuses dont 1% sont représentés par les angiomyolipomes épithélioïdes rénaux (AMLeR). Il existe une forme sporadique et une autre forme associé avec la sclérose tubéreuse de Bourneville (STB). Vu leur aspects cliniques et surtout radiologiques, les AMLeR, sont fréquemment confondus avec un carcinome à cellules rénales (CCR). Le diagnostic est essentiellement histologique se basant sur l'immunohistochimie. Les AMLeR à potentiel agressif et les AMLeR malins ont une évolution locorégionale, ganglionnaire ou métastatique qui peut conduire au décès. Les modalités thérapeutiques varient en fonction de la présentation de la maladie.

## Patient et observation

**Figure 1:**
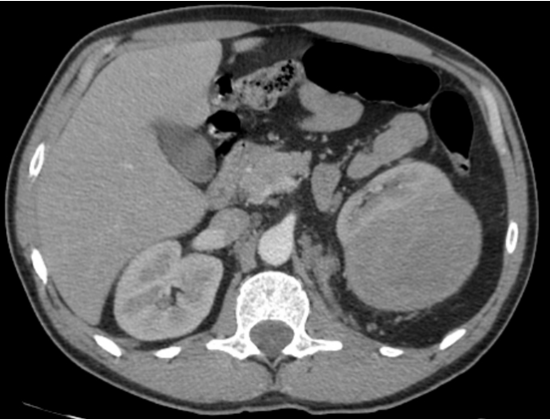
Masse tissulaire médio-rénale gauche de rehaussement hétérogène après PDC associés à des adénopathies lomboartique

**Figure 2:**
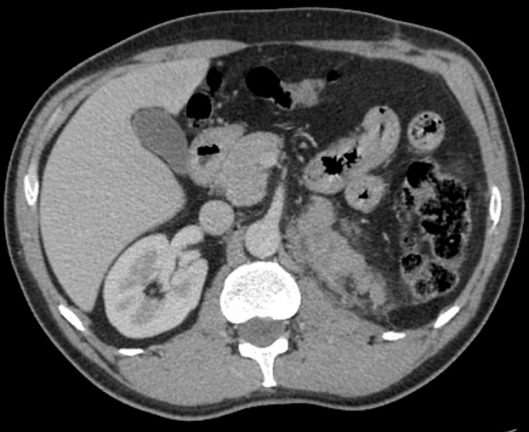
Coulée ganglionnaire lomboaortique

**Figure 3:**
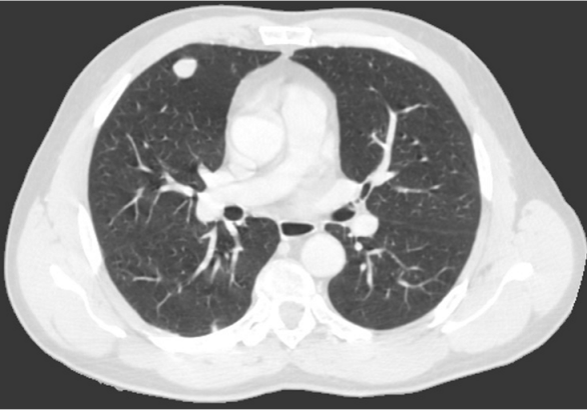
Métastase pulmonaire du lobe moyen

**Figure 4:**
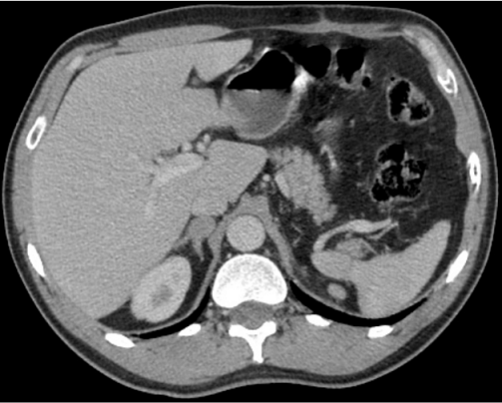
Métastase du corps de la surrénale droite

Il s'agit de Mr R A., âgé de 51 ans, traité en 2011 pour une lithiase rénale gauche par lithotritie extracorporelle, qui consulte pour des lombalgies gauche associée à une altération de l’état général à type d'amaigrissement, d'asthénie évoluant depuis 3 mois sans notion d'hématurie. L'examen clinique était sans anomalies et le bilan biologique trouvait une fonction rénale correcte (créatininémie à 74.5 µmol/l), une numération formule sanguine était normale et un ECBU était stérile. L’échographie abdominale trouvait une masse tissulaire hypoéchogéne de 12 cm rénale gauche. Le scanner thoracoabdolminopelvien montrait une volumineuse masse tissulaire médio-rénale gauche de 13.5 cm de grand axe à développement exorénal hétérogène se rehaussant après injection du produit de contraste (PDC), évoquant en premier un carcinome rénal (Figure 1) associée à une infiltration de la graisse péri rénale avec des ganglions lombo-aortiques gauches d'allure suspecte et absence de localisation secondaire hépatique ou pulmonaire. Le patient a été opéré il a eu une néphrectomie totale élargie réalisée par voie sous costale avec des suites post-opératoires simples et un court séjour hospitalier de 5 jours. L’étude anatomopathologique de la pièce opératoire a trouvé une tumeur largement nécrosée qui comporte des zones charnues de couleur blanc grisâtre et indurées. Cette tumeur infiltre le sinus. A l’étude microscopique les cellules tumorales sont de grande taille, de forme variable, tantôt d'aspect épithélioide et tantôt d'aspect rabdoide. De nombreuses figures de mitoses atypiques (6 mitoses/ 10GC) sont observées ainsi que la présence de nombreux embols tumoraux. La nécrose tumorale est estimée à 20%. L’étude immuno-histochimique sur la coupe de parrafine était positive pour EMA, HMB45 et Mélan A. Toutes ces constations ont permis de confirmer le diagnostic d'angiomyolipome épithéloide monophasique ou pécome malin classé pT2 N1 M0. Le suivi post-opératoire à 3 mois, trouve un patient en bon état général, asymptomatique avec une fonction rénale normale. Un scanner de contrôle à 6 mois a montré une progression en taille et en nombre des adénomégalies partiellement nécrosées lombo-aortiques et iliaques primitives gauches (Figure 2) étendues à la loge de néphrectomie gauche avec apparition des localisations secondaires pulmonaires (Figure 3) et surrénalienne droite (Figure 4). Le dossier a été discuté en RCP et un traitement type inhibiteur de mTOR a été démarré. Après 6 mois de thérapie ciblé, le patient se porte bien avec une stabilisation des lésions secondaires à l'imagerie.

## Discussion

L'angiomyolipome (AML) est une tumeur rénale bénigne, qui représente environ 3% des masses solides du rein, formé de trois composantes tissulaires à des proportions variables: vaisseaux anormaux, cellules musculaires lisses et tissu adipeux La variante épithélioïde est plus rare correspondant à moins de 1% de l'ensemble des angiomyolipomes [1]. Les angiomyolipomes épithélioïdes rénaux (AMLeR) est une tumeur rare mésenchymateuses rapportée pour la première fois par Mai et al. [2] en 1996. En 2004, l′Organisation mondiale de la santé (WHO) a classé l'AMLeR comme une tumeur mésenchymateuses ayant un potentiel malin et faisant partie de la famille des Pecomes pour perivascular epithelioid cells. L’âge moyen de découverte est de 40 ans avec des extrêmes de 14 à 70 ans [3]. Dans certain cas il est difficile de différencier les AMLeR des autres tumeurs rénales solides telles que les oncocytome, les carcinome à cellules rénales et les lésions sarcomateuses en se basant seulement sur l′imagerie. La TDM ou l'IRM sont fréquemment utilisées pour détecter les foyers de graisse qui caractérisent l'angiomyolipome. Cependant, Aucun critère de densité ne permet d’établir le diagnostic sûr d'AMLeR classique [4]. Dans notre cas, en raison de la suspicion de carcinome une néphrectomie a été pratiquée. Histologiquement, la tumeur est essentiellement composée de cellules épithélioïdes, alors que dans certains cas, il peut montrer des similitudes avec l'AML. L immunohistochimie joue un rôle clé dans le diagnostic différentiel. Les AMLeR sont typiquement marqués par les anticorps anti-HMB45 et Melan A [5] mais le marquage est parfois très focal, limité à quelques cellules tumorales en particulier autour des vaisseaux sauf dans les secteurs épithélioïdes où le marquage est diffus. Dans notre cas, la coloration immunohistochimique était positive pour EMA, HMB45 et Mélan A. Les AMLeR peuvent récidiver localement voir métastaser dans le foie, les ganglions, les poumons ou l'os [6]. Le pourcentage d’évolution défavorable est cependant très variable dans la littérature. Les facteurs de risque identifiés sont: une taille tumorale supérieure à 7 cm, une morphologie épithélioïde à plus de 70%, une activité mitotique > 2 mitoses pour 10 grands champs, la présence de mitoses atypiques, l'extension extra-rénale ou de la nécrose [6]. Sur la pièce de néphrectomie de notre patient il y avait 4 facteurs de risque de récidive locale ou de métastase à distance. La néphrectomie totale élargie devrait être indiquée pour les AMLeR malins avec envahissement locorégional ou métastatique. Le traitement adjuvant ou de première ligne à base de chimiothérapie ou de thérapie ciblée doit être discuté en RCP [7].

## Conclusion

L'oncogenèse des AMLeR sporadiques ou associés à la STB est lié au gène TSC2 qui active la voie des mTOR. Le diagnostic est anatomopathologique. Avec quatre paramètres (clinique, TDM, anatomopathologique et bilan d'extension), on confirme le diagnostic d'AMLeR à potentiel agressif et AMLeR malin. Pour ce type de tumeur, le traitement est celui d'un CCR. La thérapie ciblée est de plus en plus entrain de prouver son indication dans l'AMLeR.
